# A Wireless Multi-Channel Recording System for Freely Behaving Mice and Rats

**DOI:** 10.1371/journal.pone.0022033

**Published:** 2011-07-12

**Authors:** David Fan, Dylan Rich, Tahl Holtzman, Patrick Ruther, Jeffrey W. Dalley, Alberto Lopez, Mark A. Rossi, Joseph W. Barter, Daniel Salas-Meza, Stanislav Herwik, Tobias Holzhammer, James Morizio, Henry H. Yin

**Affiliations:** 1 Department of Psychology and Neuroscience, Department of Neurobiology, Center for Cognitive Neuroscience, Duke University, Durham, North Carolina, United States of America; 2 Behavioural and Clinical Neuroscience Institute and Department of Experimental Psychology, University of Cambridge, Cambridge, United Kingdom; 3 Department of Microsystems Engineering (IMTEK), University of Freiburg, Freiburg, Germany; 4 Howard Hughes Medical Institute Janelia Farm Research Campus, Ashburn, Virginia, United States of America; 5 Department of Psychiatry, University of Cambridge, Addenbrooke's Hospital, Cambridge, United Kingdom; 6 Triangle BioSystems, Int'l, Durham, North Carolina, United States of America; University of Chicago, United States of America

## Abstract

To understand the neural basis of behavior, it is necessary to record brain activity in freely moving animals. Advances in implantable multi-electrode array technology have enabled researchers to record the activity of neuronal ensembles from multiple brain regions. The full potential of this approach is currently limited by reliance on cable tethers, with bundles of wires connecting the implanted electrodes to the data acquisition system while impeding the natural behavior of the animal. To overcome these limitations, here we introduce a multi-channel wireless headstage system designed for small animals such as rats and mice. A variety of single unit and local field potential signals were recorded from the dorsal striatum and substantia nigra in mice and the ventral striatum and prefrontal cortex simultaneously in rats. This wireless system could be interfaced with commercially available data acquisition systems, and the signals obtained were comparable in quality to those acquired using cable tethers. On account of its small size, light weight, and rechargeable battery, this wireless headstage system is suitable for studying the neural basis of natural behavior, eliminating the need for wires, commutators, and other limitations associated with traditional tethered recording systems.

## Introduction

A major goal of neuroscience is to elucidate how behaviour is generated by the nervous system. Reaching this goal requires large-scale recording of neural activity in multiple structures during natural behaviour in freely moving animals. Traditional single-electrode approaches usually involve restrained or head-fixed monkeys performing artificial behavioral tasks [1], [2]. This approach suffers from two major limitations: (1) often only one neuron can be recorded at a time, with a strong bias to select cells showing burst firing patterns at the time of selection; and (2) the animal's behavior is severely restrained, causing considerable discomfort and making it nearly impossible for the learning and performance of even the simplest behavioral tasks. Consequently, little progress has been made in understanding the neural mechanisms of behavior using the traditional single-unit approach.

Recent advances in electrode array technologies address the first of these limitations, making it possible to use multiple chronically implanted electrodes to measure neural activity from multiple brain regions and neurons simultaneously [3], [4], [5], [6], [7]. With the development of miniaturized microarrays and headstages, investigators can record from up to hundreds of neurons in the brains of freely moving rodents, in many cases for as long as virtually the entire adult lifespan of the animals [8], [9], [10], [11]. Despite the power of the multi-channel arrays, their full potential is limited by the need to use a tethered setup: since the electrode array is connected with a headstage, which is then connected to a data acquisition system via wires, these wire tethers can restrict the movements of the animals. Very often during a recording session unrestrained animals will attempt to remove and bite the connectors and cables, causing costly damage. In addition, it is often necessary to use a commutator to prevent tangling of the wires due to the rotation of the animal's body, which may introduce additional noise to the system. With continuing advances in electrode technology offering ever increasing channel counts, the corresponding increase in the number of wires connected to the animal exacerbates the problem. Although the use of multiplexing systems can offset increases in the number of wires needed, it cannot eliminate wires altogether.

To overcome the limitations associated with tethered recording, we have developed and tested a wireless headstage system, replacing the wires with a radio transmitter system. This light-weight, compact wireless headstage is ideal for recording from small animals, in particular mice and rats which are the species of choice for behavioral testing and represent the best available animal models for neurological and psychiatric disorders. Using this wireless system, we recorded single-unit activity and local field potentials from a variety of brain structures in freely moving mice and rats during a battery of behavioural testing, including unrestrained behavior and rotarod running in mice, operant lever pressing, sleeping, and the 5-choice serial reaction time task in rats.

## Results

### Overview of single-unit recording data

**Figure 1:**
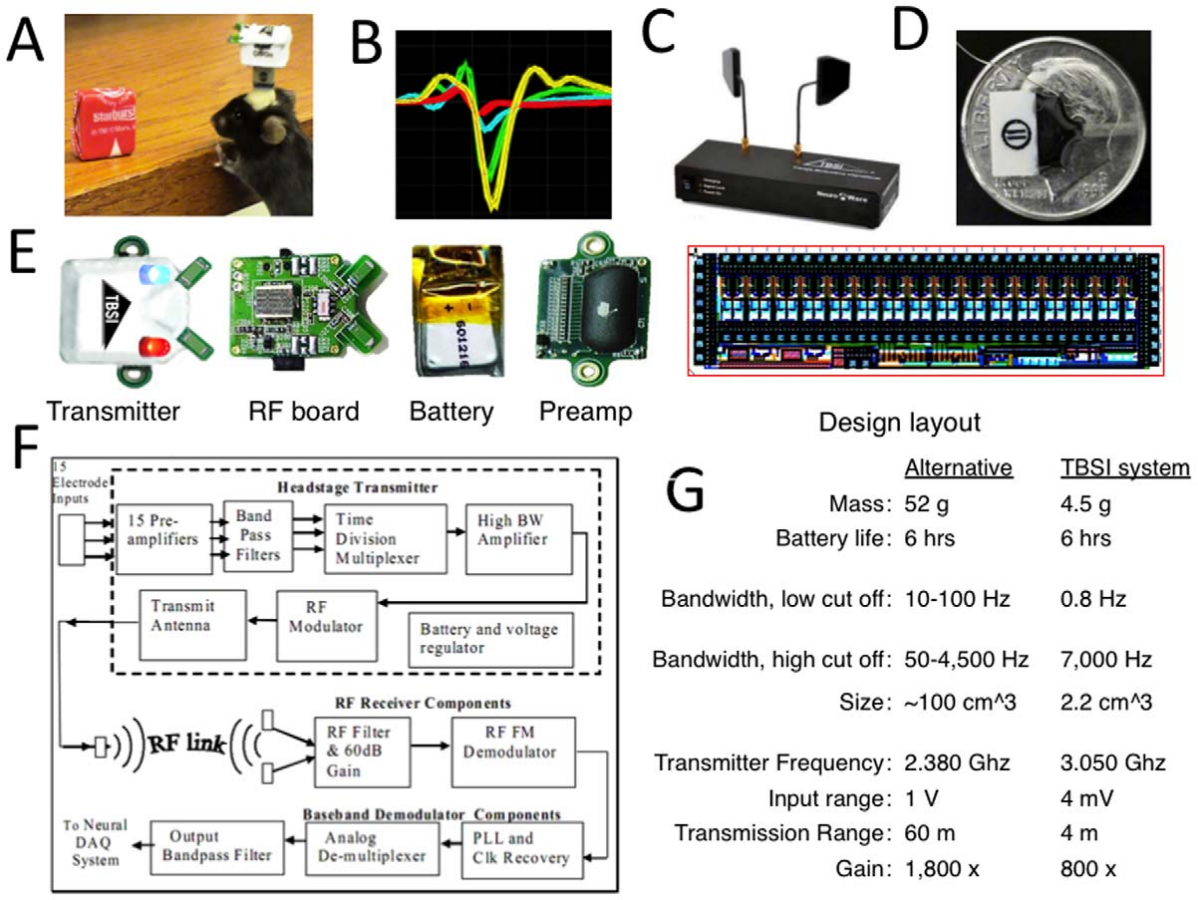
Overview of wireless telemetry system. A. The wireless telemetry transmitter is small and light enough (4.5 g) to be used in recording from small rodents, including mice, as shown here. B. An example of telemetry data recorded from a single channel. C. Receiver with dual directional antennas. D. 16-channel microwire array with 25mil Omnetics connector. E. From left to right: fully assembled transmitter with tracking LED's, the top PCB board which contains RF transmitter circuitry and antennas, the rechargeable Li-ion battery, and the bottom PCB board which contains pre-amplifier and multiplexing circuits and design layout of custom ASIC (Application Specific Integrated Circuit) which contains preamplifiers, band pass filters, and time-division multiplexing circuits. F. System diagram of transmitter and receiver. Raw neural signals are fed into the input connector of the wireless transmitter, and amplified, band-pass filtered, and multiplexed into one analog signal. This multiplexed signal is RF modulated and transmitted via two dipole antennas. After traveling to the receiver, the radio frequency signal is received by one of two antennas, filtered and gained, demodulated, then de-multiplexed. The analog receiver then takes the analog signal from each channel and filters it before sending it to an output connector. G. Comparison between our TBSI wireless system and another currently available wireless system developed by Szuts et al [31]. Our system is much smaller and lighter than the alternative. To our knowledge, it is the only system that can be used in recording from mice and other similarly sized animals.

We successfully recorded single units from the mouse dorsal striatum and substantia nigra and the rat dorsal striatum, ventral striatum and medial prefrontal cortices. The receiver can be placed up to 5 meters away from the wireless telemetry transmitter. No interference with behavior was observed in any of the tasks using the wireless telemetry system; compared with tethered recording, the animals had greater freedom of movement. A common problem in tethered recording from rodents, especially rats, is the removal and chewing of the connectors and cables, which end the recording session prematurely and result in costly damage. This problem was avoided by the wireless recording. We did not observe any animal attempting to remove the wireless headstage, presumably because it did not significantly interfere with their movements. Mice with the wireless headstage are capable of running quickly and climbing out of a cage (**Fig. 1A**). Rats performing a sustained visual attention task often reached velocities of ∼25 cm/s.

**Figure 2:**
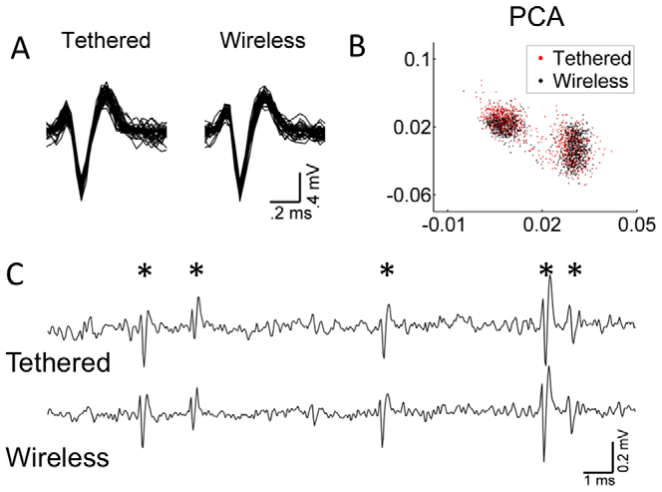
Side-by-side comparison between wireless and tethered recording systems. **A.** Simultaneously recorded action potentials from the substantia nigra using wireless and tethered recording systems in an awake, behaving mouse. The gain of both the tethered and the wireless system were set at 2×. **B.** Principal component analysis (PCA) of 30 seconds of neural data from the same recording session. The cluster on the left is the spike, while the cluster on the right is noise. **C.** Comparison of 20 ms of raw analog data, recorded from the same recording session. Asterisks mark neuron action potentials.

As shown in **Fig. 1B**, we successfully recorded from single neurons from the mouse substantia nigra while the animal is moving freely in an operant chamber. These neurons include both putative GABAergic and dopaminergic cells, as classified by their waveforms and firing properties. **Fig. 2** show raw data simultaneously recorded using a conventional tethered recording system and our wireless system. Direct comparison of the signals suggests comparable spike waveforms and noise levels: (Neuro16G2's RMS noise is 6.2 uV, while that of the wireless system is 10 u).

### Activity of the dorsal striatum in mice during rotarod test

**Figure 3:**
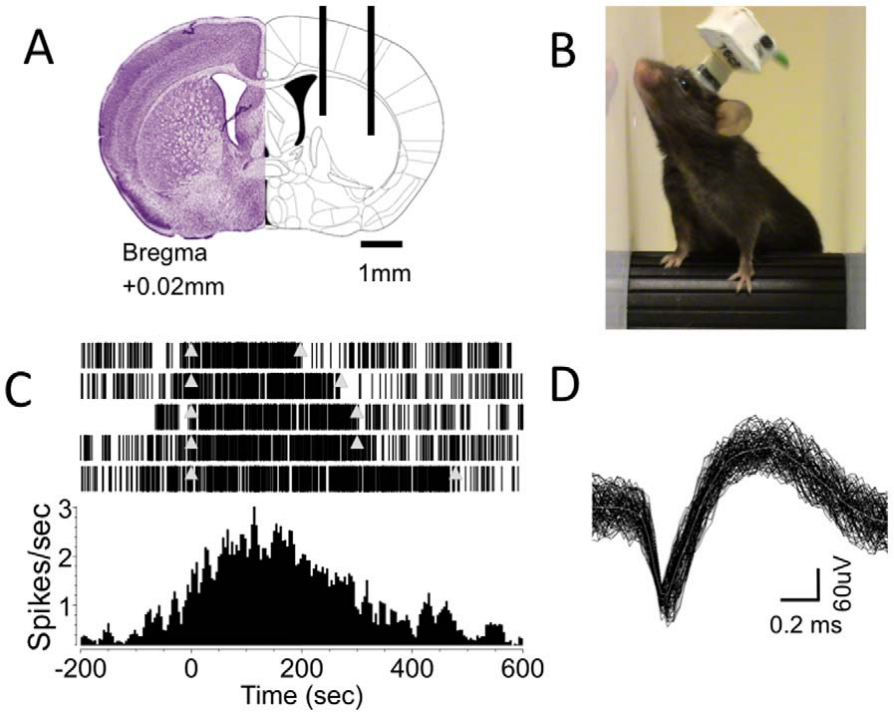
Wireless recording of single-unit activity from the mouse dorsal striatum during rotarod running. **A.** Coronal section of a mouse brain showing superimposed electrode placement targeting the dorsal striatum. This experiment used a 2 by 8, 16 channel microwire electrode array. **B.** Photo of a mouse during a rotarod task. The wireless telemetry system allows the animal to fall and rest between trials without getting wires tangled up in the testing apparatus, yet is small and light enough that it minimally interferes in the task itself. **C.** Raster plot and peri-event time histogram of a putative medium spiny projection neuron from the dorsal striatum. The start and end of each trial are marked by triangles. Notice the clear difference in firing rates between the trial state and the resting state. The histogram bin size is 4 seconds. **D.** Action potential waveform of the neuron shown in C.

**Fig. 3C** shows a raster plot and peri-event time histogram of a task-related neuron from the dorsal striatum. The firing rate of this putative medium spiny projection neuron increased during the trial and decreased during rest periods. It should be noted that the mouse was usually quite active during rest periods. Thus the spiking of this neuron is not just correlated with general motor output, but is specific to running on the rotarod, in agreement with similar data reported by previous studies [8], [9].

### Activity of the dorsal striatum in rats performing the force-sensitive lever task

**Figure 4:**
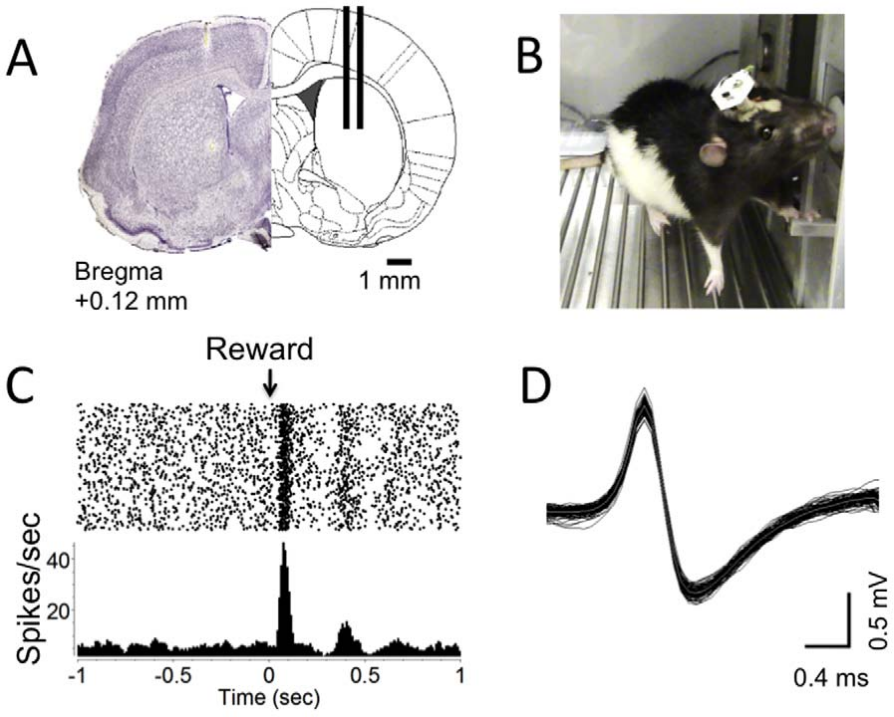
Wireless recording of single unit activity from the rat dorsal striatum during operant conditioning. **A.** Coronal sections of a rat brain showing the striatum and the placement of the microwire arrays. A 16-channel, 2×8 microwire array was used. **B.** Rat performing operant task of pressing a small protrusion in order to receive a reward. The lever measures the force that is exerted upon it, and the reward is contingent upon the duration and force of the press. Use of the wireless telemetry system for this facilitates task acquisition and prevents the rat from chewing removing the headstage and chewing the wires. **C.** Raster plot and peri-stimulus time histogram of a putative tonically active cholinergic neuron recorded from the rat striatum. Bin size for the PSTH is 20 ms. The X-axis shows time from the delivery of a food pellet reward following a successfully completed lever press. The neuron showed burst firing immediately after reward delivery. **D.** Action potential waveform of the neuron shown in C.

We also recorded from the dorsal striatum in a rat during operant lever pressing. After one session of training, the rat learned to press a force sensitive lever and exert the required force needed to earn rewards. The wireless system reliably recorded task-related single units in the striatum (**Fig. 4a**). **Fig. 4B** shows the rat performing the operant task, and **Fig. 4C** shows a striatal neuron showing bursting activity in response to an auditory stimulus indicating the delivery of a food pellet reward. This neuron is classified as a putative giant cholinergic neuron [12], based on its wide spike duration and a tonic level of baseline firing rates of 4–5 Hz (**Fig. 4C** **and** **Fig. 4D**).

### Multi-structure wireless recording of LFP signals and single units in rats performing the 5-CSRTT

**Figure 5:**
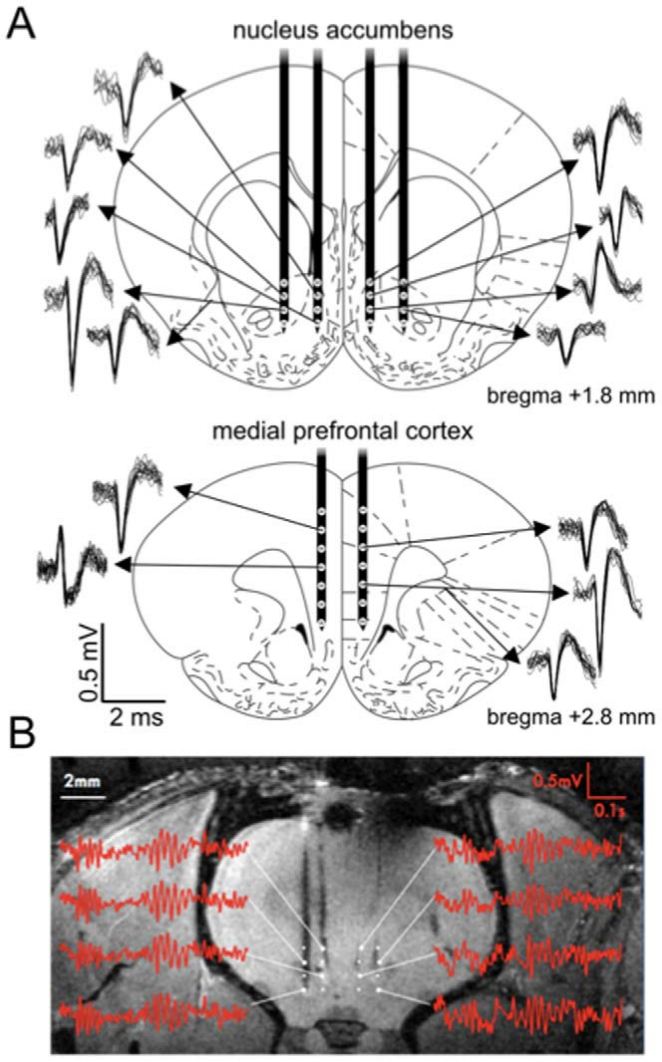
Neuronal ensemble recording in multiple structures on both sides of the brain in awake rats. **A.** Representative stereotaxic atlas images of the ventral striatum (*upper panel*) and prefrontal cortex (*lower panel*) show the approximate location of our silicon probe arrays. Individual electrodes are represented by circles on each of the black shafts. A selection of superimposed spike waveforms are drawn beside each image with arrows corresponding to the recording location of unit. **B.** Structural MRI taken following fixation of the brain and removal of probes. Local field potentials were recorded bilaterally from 16 sites in the ventral striatum (signals from 8 are highlighted). Recordings from both ventral striatum and prefrontal cortex (not shown) were characterised by prominent gamma-55 oscillations easily visible in the raw signal.

**Figure 6:**
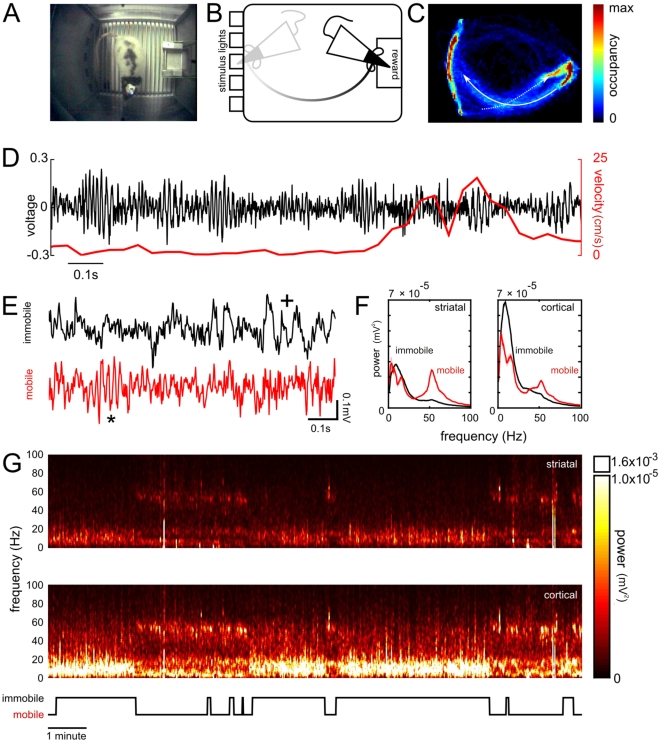
Wireless recording combined with motion tracking during active behaviour and sleep. **A.** The wireless telemetry unit allowed the animal to move freely in the operant chamber. The receiver antenna is partly visible in the lower left of the frame (note that this was kept outside of the area used for motion tracking). **B.** Following a nose poke to one of five recessed apertures (stimulus lights), the animal moves quickly to collect the food reward at the rear of the chamber; such fast movements were not impeded by the presence of the wireless headstage. **C.** Occupancy map of the operant chamber floor area with hotter colors indicating more time spent in a region. The high occupancy area on the left reflects the time the animal spends during sustained attending to the stimulus lights, involving fast movements of the head. This map also highlights the different routes taken by the rat on its outward (solid arrow) and return trips (dotted arrow) from the food tray. **D.** Wide band LFP signal from an example striatal electrode. Despite the fast movements, the LFP signals are free from movement-induced artifacts; gamma oscillations are seen throughout the changes in velocity. **E.** The change in the spectral composition in the PFC and NAc between mobility and immobility can be seen in the wide-band LFP. Gamma-55 events (*) are absent during sleep, defined by periods of immobility which is dominated by lower frequencies (+). **F.** Power spectral estimates reveal that the increase in slow oscillations characteristic of slow wave sleep is more prominent in cortical sites than striatal sites. **G.** Spectrograms during a session in which the animal alternated between putative wakefulness and sleep. The lower ethogram shows when the animal was classed as mobile and immobile, using motion tracking.

When coupled to our custom-made silicon probe arrays in awake rats, we were able to the record the activity of the PFC and NAc sub-regions on both sides of the brain. Example single unit recording is shown in **Fig. 5A**, which illustrates a selection of neurons in an awake rat. In this example, multiple spike waveforms were isolated on the same electrode in two cases, giving a total yield of up to 14 units spread across the PFC and NAc in both hemispheres. We also recorded LFP signals from all 30 sites; in agreement with previous studies in the ventral striatum of awake rats [13], [14], [15] our LFP signals were punctuated by bouts of high amplitude gamma-band oscillatory activity. These oscillations were strongly harmonic with a frequency centered around 55 Hz and typically lasting 50–200 ms (**Fig. 5B** **&** **Fig. 6D**). Gamma-band oscillations were accompanied by prominent activity at other frequencies including <10 Hz, ∼20 Hz and 80–100 Hz, broadly in agreement with previous studies [13], [14].

### Motion tracking using a wireless headstage

Rats rapidly shuttled back and forth between the stimulus lights and the food magazine to collect rewards, on average completing over 100 trials in a 30 minute period. We used LEDs on the headstage (red and blue; see **Fig. 6A**) to track the motion of the rats during task performance. Analyzed tracking data were used to construct the spatial occupancy map illustrated in **Fig. 6B** **and** **Fig. 6C**; this particular rat showed a marked preference for right-handed turns when beginning trials (i.e. from rewards to stimulus lights) in contrast to left-handed turns in order to collect rewards, whilst other rats showed idiosyncratic movement preferences (not shown). Despite the fast movements of the rats, which often reached a velocity of ∼25 cm/s, we were able to record high quality data without artifacts (**Fig. 6D**).

### Recording during sleep

In 3 rats, we also recorded brain activity during periods of quiet rest in which rats were allowed to behave freely in the dark. Extended periods of immobility were accompanied by a distinct shift in the LFP activity away from gamma-band activity toward large amplitude, lower frequency oscillations (**Fig. 6E & F**). During these periods, power spectral analysis revealed the suppression of gamma-band activity in both NAc and PFC regions during sleep, showing an abundance of low frequency oscillations characteristic of NAc activity during slow wave sleep [16], [17]. The rapid switching of network oscillatory states during sleep and wakefulness can be seen in the spectrograms plotted in **Fig. 6G**.

## Discussion

Our data demonstrate the viability of the wireless headstage system, which promises to be a useful tool for future research on the neural substrates of behavior. It can be used to record both single unit and local field potential activity, and the quality of the wireless recording appears to be comparable to that of the tethered recording system (**Fig. 2**). Therefore, under a variety of conditions tested in our experiments, the wireless recording system can be used to replace the tethered system.

### Single unit recording from mice and rats

The substantia nigra is a region in the midbrain containing the widely studied dopamine cell groups, which project mainly to the striatum. Studies have shown the critical importance of the nigrostriatal circuit in voluntary behavior [18], [19].

As shown in **Fig. 1B**, the single unit isolation is excellent when we recorded from the nigral neurons during free behavior in an operant chamber. Likewise, we were also able to record from the medium spiny projection neurons from the dorsal striatum during rotarod running (**Fig. 3**). We replicated the task-related single-unit neural activity previously observed with tethered recordings [8], [9].

Using chronically implanted microarrays, we recorded from the sensorimotor striatum of rats performing a force-sensitive lever pressing task. As shown in **Fig. 4**, we were able to record high-quality single unit data using this task. In traditional tethered recording, rats tend to pull and bite the cables connecting their headstages to the data acquisition system. This problem is eliminated by the use of the wireless system. Although we only used a 16-channel array, there is no question that rats will be able to bear the weight of much larger headstages (e.g. 64 channels).

### Multi-site single unit and local field potential recording from rats

Studies in both humans and animals have uncovered a growing body of evidence that fronto-striatal circuits play a major role in the regulation of impulsive behaviour. Lesions of the nucleus accumbens (NAc) and the prefrontal cortex (PFC) in rats produce profound changes in specific measures of impulsivity [20], [21], [22]. We used multi-site electrophysiological recording to assess activity in the PFC and NAc during performance of the 5-choice serial reaction time task - a test of sustained visual attention and impulsivity [23]. Previous studies of gamma-band oscillatory activity in the ventral striatum of awake behaving rats have linked activity in this range to aspects of reward processing [14].

Using silicon probes, we were able to record neural activity from two brain regions simultaneously using 32 channels. To our knowledge, this is the first report of multi-site silicon probe arrays being used with wireless telemetry. A variety of neuronal types are included in this dataset (**Fig. 5A**), including putative cortical pyramidal cells, accumbens medium spiny neurons and fast-spiking interneurons, which can be classified by their firing properties and spike waveforms [16].

We also used the wireless system with LED lights to record brain activity while tracking the motion of the animal in the operant chamber during the performance of a task measuring sustained visual attention. In addition to recording during task performing, we were also able to acquire data from rats during brief periods of sleep characterized by a profound shift in network activity away from prominent gamma-band activity toward lower frequency activity.

### Comparison with currently available wireless systems

A satisfactory wireless recording system should meet three major criteria. 1) it must be compact and light weight, without sacrificing the number of recording channels, so that the animal can wear it while moving freely; 2) the signal acquired with the wireless system should be comparable to that from tethered recording; and 3) the system should have sufficient range so that animals can move far away from the receiver without reducing the quality of the recording.

Recently, several wireless recording systems have been introduced, but none have met all of the above criteria [24], [25], [26], [27], [28], [29], [30]. Prior to the present report, perhaps the best example of the wireless telemetry system was described in a recent report [31]. As shown in **Fig. 1**, in comparison with the system introduced by Szuts et al., our system is superior in most respects. The sole exception is the range of the wireless signal. The system introduced by Szuts is shown to have a range up to 60 m, making it suitable for recording neural activity outdoors and in tunnels. However, the range for our system (∼5 m) is sufficient for all known types of behavioral experiments performed indoors. The system introduced by Szuts et al is much larger and heavier, making it unsuitable for use in mice (**Fig. 1G**). At 45 g, it is heavier than the average mouse (∼20–30 g) and other comparable small animals such as zebra finches (10–15 g). It is therefore more suitable for recording from rats and similarly sized animals outdoors in the natural environment, whereas our system is more suitable for recording indoors from rats, mice, and other small animals. The requirement of a backpack harness to carry the power source for the Szuts system and other existing systems [28] also requires time for animals to become habituated to wearing the harness, and its presence may impede movement, whereas in our system, backpacks are not required as the power source is carried on the animal's head. As most neuroscience experiments are carried out in the laboratory rather than in the field, our system may be more suitable for the needs of most researchers. Nonetheless, depending on one's needs, a choice can be made between the two wireless systems.

### Implications for future research

Behavioral studies often require many trials as well as stability in animals' performance. Our system removes potential distractions and mechanical disturbance from cables, allowing animals to move quickly in execution of the task. It is not sensitive to movement artifacts–a common type of signal contamination in tethered recordings. In tethered recording setups, potential sources of noise may come from the movement of suspended cables through the environment and from mechanical stress transmitted to head-stage connectors. These sources of noise are minimized in our wireless recording system. The low power demands of the wireless headstage also support the inclusion of the LEDs for head position tracking, which proved convenient in the elucidation of the movement patterns during the 5-choice serial reaction task.

As already mentioned, another advantage of our system is that it is small and light enough to be used in mice, which are becoming increasingly popular as the organism of choice in neuroscience, due to the large number of genetic tools available [32], [33]. Our system can also be used in other small animals such as song birds [34].

Finally, the wireless recording system can be uniquely powerful in studying social behavior, and other natural behaviours which would be difficult to study with a tethered recording device. Configured at different transmission frequencies, it could be used to record neural activity from multiple subjects during feeding, play, copulation and other social interactions. This application of the wireless technology can open up new avenues of research on the neural substrates of social behavior in small animals.

## Materials and Methods

### Ethics Statement

All procedures conducted in the USA were approved by the Institutional Animal Care and Use Committee at Duke University and followed National Institutes of Health guidelines (Protocol Number: A087-08-04), whilst those procedures conducted in the UK were approved by the local ethical review panel of the University of Cambridge and by UK Home Office regulations in accordance with the Animals (Scientific Procedures) Act of 1986 (Project Number: 80/2234).

### Wireless system

Neural signals picked up by the electrodes require analog signal processing prior to A/D conversion. Adequate gain, a wide bandwidth, and a significant channel count are necessary specifications of the signal preconditioning electronics, especially when both low frequency signals (local field potentials, LFP) and neuron spiking activity are recorded. Single unit activity is usually in the range of 300–8,000 Hz, while LFP signals are usually in the range of 0–100 Hz. Because these signals are low-voltage, early amplification is needed to overcome additive noise from transmission and the environment. Furthermore, integration and packaging of these multi-channel analog functions into a lightweight, miniaturized form factor close to the electrodes is also needed for convenient recording from small animals. The transmitter described here (Triangle BioSystems, Int'l, NC) meets these specifications (**Fig. 1G**), and is much smaller, lighter, and transmits a wider bandwidth when compared to a recently published wireless telemetry system which operates on similar principles [31].

The signal path is shown in **Fig. 1E**. Briefly, voltage fluctuations generated by neurons are picked up by implanted electrodes. The signals are first amplified with a gain of 100 and filtered with a second order cascaded bandpass architecture from 0.8 Hz to 10 kHz, providing sufficient range to capture both LFP and single-unit activity. Next, the filtered and buffered signals on each channel are multiplexed into a single output. The rate of multiplexing is 50 kHz, i.e. each channel is sampled 50,000 times per second. Depending on the number of channels configured, this could mean a clock speed of up to 3.2 MHz for a 64 channel wireless transmitter. The low noise pre-amplification of 8.2 µV RMS, buffering, and time-division multiplexing are all accomplished via a custom mixed-signal 32 channel ASIC chip design (**Fig. 1E**). The 32 channel ASIC chip size is 1.5 mm × 5.5 mm and the 15 channel ASIC size is 1.5 mm × 3.4 mm. This Complementary Metal Oxide Semiconductor (CMOS) die is fabricated using a 0.5 µm, double poly, triple level metal CMOS process and is wire-bonded and encapsulated onto a Preamp PCB board for structural integrity and signal routing (**Fig. 1E**). After the channels are multiplexed into one broadband signal, the signal is converted to a frequency modulated (FM) radio-frequency (RF) signal, via a voltage controlled oscillator (VCO) circuit on a separate RF PCB. This signal is a high frequency sinusoidal waveform centered at 3.05 GHz; its frequency varies 50 MHz in proportion to the voltage level of the input analog signal. It is fed to splitter and two dipole antennas which are orthogonally oriented to create a circular polarized optimal beam pattern (Fig. 2C). A 45 maHr Lithium Ion rechargeable battery is placed between the RF PCB and Preamp PCB to provide 6 hours of recording time (**Fig. 1E**). The total headstage weight is 4.5 grams.

The RF signal is carried through various non-ferrous materials, e.g. air, glass, wood, plastic, etc. It can bounce off metal and is transmitted through walls. Due to its FM characteristics, this robust signal is resistant to distortions. The power of the signal can be adjusted, and broadcast in a 4 m radius about the transmitter. It is picked up by the receiver, which is connected to the data acquisition system (**Fig. 1C**). The use of two antennas on the receiver allows a best-signal received power switching scheme, which rapidly chooses an antenna from which to extract the signals. Because this switching occurs at a much higher rate than the sampling of the multiplexer, no gaps in the data are created when the receiver switches from one antenna to the other. Finally, the RF signal is filtered, demodulated, then demultiplexed into individual channels using a edge sensitive clock recovery and sampling scheme. The analog output section of the receiver provides additional 4^th^ order bandpass filtering of 0.1 Hz to 7 KHz per channel with a low impedance output driver before they are sent to a data acquisition system.

### Animals

Data were obtained from two male mice (C57Bl6/J) weighing between 25 and 35 g (Jackson Laboratory, ME), one Long-Evans rat weighing 400 g at the start of the experiments (Charles River, MA), and 8 Lister Hooded rats (∼420–460 g; Charles River, UK).

### Surgery and histology

In all experiments recording single unit data (Duke group), 16-channel microwire arrays (**Fig. 1D**, Innovative Neurophysiology, NC) were used. The arrays consist of micro-polished tungsten wires, 50 um in diameter, arranged in a 2×8 configuration (Fig. 1). Row spacing was 200 um and pitch was 150 um. The microwires were attached to an Omnetics connector (Omnetics Connector Corporation, MN). The length of the wires range from 5 mm to 7 mm depending on the structure targeted. A 16-channel array weighs ∼0.26 g, a 16-channel headstage for tethered recording weighs ∼0.93 g, and the wireless headstage weighs ∼4.5 g (see **Fig. 1**).

The animals were anesthetized with isofluorane, and placed in a stereotaxic frame. After creating a craniotomy (approximately 1 mm by 2 mm for each array), the arrays were lowered into the brain at the following coordinates (all in relation to bregma): mouse striatum, 0.0 mm anterior, 2.0 mm lateral, and 2.5 mm below brain surface; mouse substantia nigra, 3.0 mm posterior, 1.0 mm lateral, and 4.6 mm below brain surface; rat striatum 0.1 mm anterior, 4.0 mm lateral, and 4.5 mm below brain surface [35], [36]. Animals were allowed to recover for 1 week before any testing or training began.

At the end of the experiment, all animals were sacrificed and perfused transcardially with 0.9% saline followed by 4% paraformadehyde. The placement of the electrodes was verified post-mortem after perfusion, overnight post-fixation with 4% paraformadehyde, using Nissl staining of 80-um brain slices.

### Data acquisition and analysis

Single-unit activity was recorded using the Cerebrus data acquisition system (Blackrock Microsystems, UT), along with software for viewing waveforms online during recording sessions. The data were sampled at 30 kS/s, after filtering with both analog and digital bandpass filters (analog highpass 1^st^ order butterworth filter at 0.3 Hz, analog lowpass 3^rd^ order butterworth filter at 7.5 kHz, digital highpass 4^th^ order butterworth filter at 250 Hz).

### Simultaneous wireless and tethered recording

To assess the wireless telemetry system, we recorded neural activity from the substantia nigra of a mouse during free behavior using a traditional tethered system (16-channel miniaturized headstages from Blackrock Microsystems, UT) and the wireless system simultaneously. We made a custom y-connector to be able to plug in both a 16 channel tethered headstage with gain of 2 (Neuro16G2, Triangle BioSystems, NC) and a 16-channel wireless transmitter.

### Single unit recording from mouse substantia nigra and striatum during rotarod running

A computer-controlled rotarod (ENV-575M, Med Associates, VT) was accelerated from 4 to 40 rotations/min over the course of 300 seconds, and the trial ended when the mouse fell off into a holding pen below. The mouse was given 5 minutes to rest in the holding pen, then another trial was started. Use of the wireless system allowed for undisturbed resting between trials, facilitated simple trial initiation, and reduced the chances of entanglement or injury due to tethered wires. Trial initiation and cessation were electronically recorded via key stroke and saved with the neural data for analysis.

### Single unit recording from rat striatum during operant lever pressing

We also recorded from a rat while it preformed an operant behavioral task in which it was required to press a 0.5 cm × 0.5 cm × 2 cm force-sensitive lever (ENV-118, Med Associates Inc.) in order to obtain food pellets as a reward (45 mg food pellet, Bio-Serv, NJ). The operant contingency was to press the lever with a force of 30 g or more for a duration of 5 seconds or longer, in order to earn the reward The lever itself is immobile, but a sensor attached to the lever detects the applied downward force and converts this force to a voltage, which is then recorded with a data acquisition card (NI-USB-6211, National Instruments Corporation). After implanting a 16 channel multi-electrode array into the rat dorsal striatum, we trained the animal daily for 1 week on this task before recording.

### LFP and single unit recording from rat prefrontal cortex and ventral striatum

Rats were anesthetized with isofluorane and placed into a standard stereotaxic frame. Bregma was exposed and a small craniotomy was made rostral to this landmark. We implanted rats with custom silicon-based electrode arrays (IMTEK, Germany) to target the medial prefrontal cortex (PFC; bregma +2.8 mm, ∼0.7 mm lateral, 2–5.5 mm deep) and ventral striatum (NAc; bregma +1.8, 0.8 & 1.8 mm lateral, −6.5–−8.5 mm deep) on both sides of the brain. Our probes were designed to record from the anterior cingulate, prelimbic and infralimbic cortices, alongside the core and shell sub-regions ofthe NAc. To achieve this, PFC probes had 2 shafts with 7 electrodes distributed at a pitch of 0.6 mm whilst NAc probes comprised 4 shafts each with 4 electrodes at a pitch of 0.4 mm, providing a total channel count of 30 spread across both sides of the brain (see Fig. 5A). The silicon probe arrays were connected via highly-flexible polyimide (PI)-based ribbon cables to custom printed circuit boards to record local field potential activity (LFP) and single units; data were sampled (Sciworks; Datawave Technologies) at 1.5 kHz (LFP) and/or 25 kHz (single units) and filtered: 1–500 Hz for LFP or 500 Hz–10 kHz for single units. The fabrication process of these silicon-based probes and the interconnecting PI cables is described previously [37].

Prior to surgery animals had been trained on the 5 choice serial reaction time task, a commonly used task in the study of sustained attention and impulsivity in rodents [23]; the ventral striatum and medial prefrontal cortex are key structures involved in the impulsive behavior measured during this task. Briefly, the behavioral task requires animals to detect a brief (0.5 s) stimulus light in one of five nose recessed apertures. Nose pokes to the illuminated aperture are rewarded with the delivery of a food pellet (Noyes dustless pellets, 45 mg; Sandown Scientific, Middlesex, UK) to a food tray protruding from the rear of the chamber. Responses made before the stimulus light or to the incorrect aperture as well as the omission of a response within a certain time window were punished with a short timeout during which the house light was extinguished. We recorded local field potentials in these structures during task performance and baseline sessions in the dark using the wireless headstage. Testing was performed in operant chambers (25×25×25 cm, Med Associates, VM USA) housed in sound attenuating chambers. The receiving unit was placed atop the chamber. Reconstructed neural signals were amplified and filtered (AM4000; AM systems WA, USA) prior to analogue to digital conversion at 1.5 KHz with a PC running the acquisition software (Sciworks; Datawave Technologies Co, USA). The same program also recorded a 25 Hz video signal from a camera mounted in the chamber as well as behavioural events such as nose-pokes and reward deliveries.

### Motion tracking

Motion tracking data were obtained using two colored LEDs on the wireless headstage. Co-ordinates for each LED per video frame were generated using a colored spot follower in our acquisition software (Sciworks, DataWave Technologies, CO, USA). When the software occasionally failed to detect unambiguously, the LEDs coordinates were interpolated from neighboring frames. Velocity was calculated by the net displacement in a sliding window of 0.2 s. We used the motion tracking data to assign periods in which the animal's speed did not exceed 3 cm/s for 10 s as immobile (putative sleep) states.

### Statistics and data analysis

We sorted single-unit spikes (**Fig. 1B**) and analyzed the data using commercially available software (Offline Sorter, Plexon, TX, USA, and Neuroexplorer, Nex Technologies, MA, USA, MATLAB, MathWorks, MI, USA). Automatic thresholding was used to separate spike signals from noise - raw data was filtered with a 300 Hz high pass filter to remove LFP and DC offsets. 3D principal component analysis was used in conjunction with valley sorting classification methods to separate spikes from noise. Spectral analysis was performed in MATLAB. The open source toolbox Chronux [38] was used to calculate spectrograms with a window length of 2 s and 0.5 s steps. Power spectral density estimates were calculated by averaging data over the time domain in a manner analogous to Welch's method.
